# Development and temporal validation of a clinical prediction score for deep vein thrombosis following femoral neck fracture surgery: a retrospective cohort study with decision curve analysis

**DOI:** 10.1186/s12959-026-00898-x

**Published:** 2026-07-27

**Authors:** Dragan Nikolić, Srdjan Ninković, Viktor Till, Sanja Stojanović, Marijana Basta Nikolić

**Affiliations:** 1 Faculty of Medicine, University of Novi Sad, Hajduk Veljkova 3, Novi Sad, 21000 Serbia; 2 Clinic for Vascular and Endovascular Surgery, University Clinical Center of Vojvodina, Hajduk Veljkova 1, Novi Sad, 21000 Serbia; 3 Center for Radiology, University Clinical Center of Vojvodina, Hajduk Veljkova 1, Novi Sad, 21000 Serbia; 4 Clinic for Orthopaedic Surgery and Traumatology, University Clinical Center of Vojvodina, Hajduk Veljkova 1, Novi Sad, 21000 Serbia

**Keywords:** Deep vein thrombosis, Hip fracture, Risk prediction model, Clinical prediction model, Thromboprophylaxis, Temporal validation

## Abstract

**Background:**

Deep vein thrombosis (DVT) is a frequent complication after femoral neck fracture surgery, with reported incidence rates of 6–19% depending on detection methods. Existing risk assessment tools, such as the Caprini score, show limited discrimination (AUC 0.62–0.75) in orthopedic trauma populations. We aimed to develop and temporally validate a clinical prediction score specifically designed for DVT risk stratification in patients undergoing femoral neck fracture surgery.

**Methods:**

This retrospective cohort study included 2,241 consecutive patients undergoing femoral neck fracture surgery at a single Serbian center (January 2018–June 2024). The cohort was split temporally into a development set (*n* = 1,829; 2018–2023) and a temporal validation set (*n* = 412; January–June 2024). The primary endpoint was DVT diagnosed after surgery on scheduled bilateral duplex ultrasonography (24–48 h and day 7) or symptom-triggered imaging during 90-day follow-up. A multivariable logistic model with 12 prespecified predictors was developed and internally validated with 1,000 bootstrap resamples. Performance was assessed by AUC, calibration slope, Brier score, and decision curve analysis.

**Results:**

DVT occurred in 269 patients (12.0%). The DVT Risk Score (DVTS) incorporated 12 predictors, including age > 75 years, D-dimer > 500 ng/mL, albumin < 35 g/L, and ASA score ≥ 3. The optimism-corrected development AUC of the shrinkage-adjusted logistic model was 0.80 (95% CI: 0.76–0.84), and its temporal-validation AUC was 0.82 (95% CI: 0.77–0.87). The simplified integer DVTS had an apparent AUC of 0.81 in development and 0.81 (95% CI: 0.76–0.86) in temporal validation. Calibration slope was 0.94 (95% CI: 0.89–0.99) in validation; Brier scores were 0.087 (development) and 0.084 (validation). At the DVTS ≥ 5 threshold in the validation cohort, sensitivity was 85.4% and specificity 41.8%, with a negative predictive value of 95.6%; a higher threshold (≥ 9) yielded specificity of 81.6%. Three risk categories showed DVT incidence of 3.3% (low), 11.7% (moderate), and 29.2% (high) (*p* < 0.001 for trend).

**Conclusion:**

The shrinkage-adjusted logistic model maintained good discrimination and acceptable calibration in temporal validation, and the simplified integer DVTS retained good discrimination (AUC 0.81) and stratified patients into clinically interpretable risk categories. External validation and prospective impact evaluation are required before clinical implementation.

**Supplementary Information:**

The online version contains supplementary material available at https://doi.org/10.1186/s12959-026-00898-x.

## Introduction

Deep vein thrombosis (DVT) represents a major postoperative complication in patients undergoing surgical treatment for femoral neck fractures. Contemporary studies employing routine ultrasonographic screening report incidence rates of 6–19%, with the wide range reflecting differences in detection timing, screening protocols, and prophylaxis strategies [1, 2]. Femoral neck fractures predominantly affect older adults with multiple comorbidities, a population at increased thromboembolic risk [3, 4]. 

Current international guidelines from the American College of Chest Physicians (ACCP) and the National Institute for Health and Care Excellence (NICE) recommend risk-stratified thromboprophylaxis for orthopedic patients, yet they lack consensus on the optimal risk prediction approach for hip fracture populations specifically [5, 6]. The Caprini risk assessment model, the most widely used scoring system, demonstrates suboptimal discrimination in orthopedic trauma settings, with reported AUC values of 0.62–0.75 and limited capacity to distinguish between moderate- and high-risk patients [7, 8]. Population-specific scoring charts have recently been developed for hip fracture patients in Asian cohorts with promising results (AUC 0.78–0.82), but these have not been validated in European populations [9, 10]. 

Recent research has identified several laboratory and perioperative factors that improve prediction accuracy beyond established demographic risk factors. Elevated D-dimer levels, hypoalbuminemia, platelet indices, delayed surgery, and perioperative hemodynamic instability have all been independently associated with increased DVT risk after hip fracture [10].,11,12 However, few fracture-specific bedside models integrate these variables into a single score evaluated specifically for early postoperative risk stratification in patients undergoing femoral neck fracture surgery.

The primary aim of this study was to develop and temporally validate a multivariable clinical prediction score (DVT Risk Score, DVTS) for DVT diagnosed after surgery in patients undergoing femoral neck fracture surgery. Secondary aims were to assess discrimination, calibration, and model-based clinical utility using decision curve analysis, and to explore inter-rater reproducibility of bedside score calculation.

## Methods

### Study design, setting, and ethical approval

This retrospective cohort study was conducted at the Department of Orthopedic Surgery and the Department of Vascular Surgery, University Clinical Center of Vojvodina, Novi Sad, Serbia. Data from consecutive patients treated between January 2018 and June 2024 were analyzed. The study protocol was approved by the Institutional Ethics Committee (approval number: 75-2023) and conducted in accordance with the Declaration of Helsinki. The requirement for individual informed consent was waived due to the retrospective nature of the study and use of de-identified data. This study is reported according to the TRIPOD guidelines [11, 12]. Because this was a conventional regression-based prediction model, reporting followed the original TRIPOD statement.13.

### Participants

**Fig. 1:**
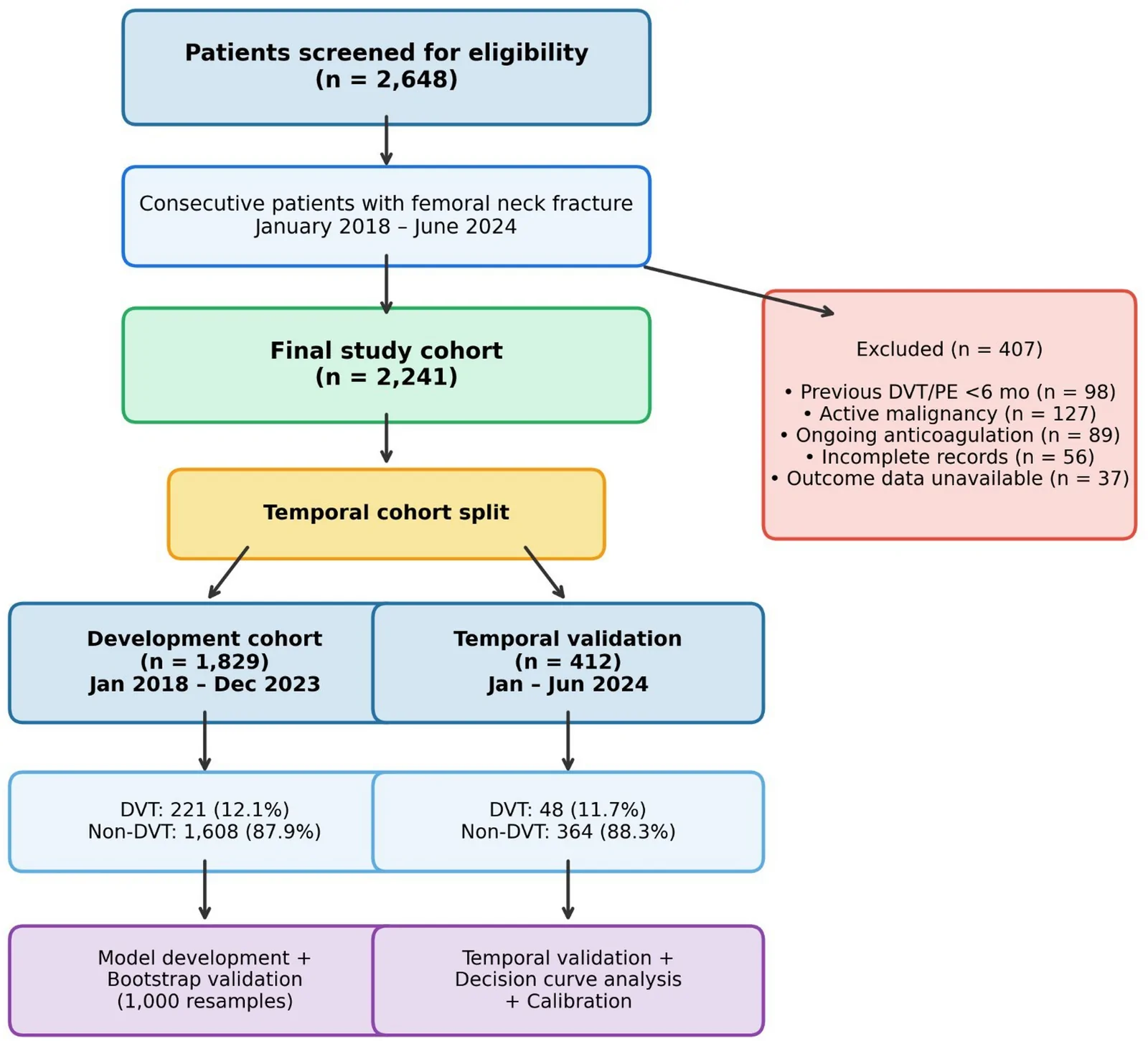
Patient selection flow diagram. Flowchart showing the screening, exclusion, and final cohort composition. DVT, deep vein thrombosis; PE, pulmonary embolism

We screened 2,648 consecutive patients who underwent surgical treatment for femoral neck fracture between January 2018 and June 2024. Inclusion criteria were: age ≥ 18 years, acute femoral neck fracture requiring surgical intervention, availability of complete medical records including laboratory and imaging data, and minimum 90-day follow-up. Exclusion criteria were: DVT or pulmonary embolism within the preceding 6 months (*n* = 98), active malignancy (*n* = 127), ongoing therapeutic anticoagulation (*n* = 89), incomplete medical records (*n* = 56), and unavailable outcome data due to transfer to another institution before 90 days (*n* = 37). After exclusions, 2,241 patients comprised the final study cohort. The patient selection process is illustrated in Fig. 1.

### Cohort split and model development strategy

In accordance with TRIPOD recommendations for temporal validation, the study cohort was divided by calendar date rather than random split.13 Patients treated between January 2018 and December 2023 formed the development cohort (*n* = 1,829), and consecutive patients treated between January and June 2024 formed the temporal validation cohort (*n* = 412). Patients treated after June 2024 were excluded from the validation cohort because the minimum 90-day follow-up required for outcome ascertainment had not been completed at the time of database lock (October 2024). Although data in the validation period were collected within routine clinical care, the validation analysis was conducted retrospectively. The cohort was split by calendar period to assess whether model discrimination and calibration were preserved in patients treated during a later time interval under the same institutional surveillance protocol.

### Outcome definition

The primary outcome was postoperatively detected DVT during surveillance after femoral neck fracture surgery, identified by bilateral lower-extremity duplex ultrasonography. Because routine preoperative ultrasonography was not performed, some early events may have represented previously undetected preoperative thrombosis [13]. Accordingly, the model should be interpreted as predicting DVT diagnosed after surgery rather than definitively incident postoperative DVT. Standardized bilateral screening ultrasonography was scheduled at 24–48 h and at 7 days after surgery; additional symptom-triggered ultrasonography was performed during the 90-day follow-up when DVT was clinically suspected. Both scheduled ultrasound examinations were completed in 2,148 of 2,241 patients (95.^8^%); the remaining 93 patients (4.^2^%) underwent only one scheduled examination (most commonly the day-7 scan was not performed due to early discharge), but all had at least one postoperative scan and remained eligible for symptom-driven assessment. All examinations followed a standardized protocol: bilateral compression ultrasonography of the common femoral, femoral, popliteal, and calf veins, with Doppler assessment of venous flow; DVT was diagnosed when the vein was non-compressible or an intraluminal thrombus was directly visualized. All ultrasound examinations were performed by experienced vascular surgeons (> 5 years of experience in vascular ultrasonography) who were not blinded to clinical data, as examinations were part of routine care. DVT was classified as proximal (popliteal vein and above), distal (below the popliteal vein, including calf veins), or bilateral according to standard diagnostic criteria [5]. 

### Thromboprophylaxis protocol

All patients received pharmacological thromboprophylaxis with low-molecular-weight heparin (LMWH; enoxaparin 40 mg subcutaneously once daily), initiated within 12 h of admission unless contraindicated by active bleeding or platelet count < 50 × 10^9^/L. Prophylaxis was continued throughout the hospital stay and for a minimum of 14 days postoperatively; patients with additional risk factors received extended prophylaxis up to 35 days at the discretion of the treating surgeon. Mechanical prophylaxis with graduated compression stockings was applied to all patients from admission, and intermittent pneumatic compression devices were used intraoperatively and for the first 48 postoperative hours. Early mobilization (weight-bearing as tolerated) was encouraged within 24 h of surgery when clinically feasible. This thromboprophylaxis protocol remained unchanged throughout the study period (January 2018–June 2024), ensuring consistent management across both the development and validation cohorts.

### Candidate predictors

A total of 25 clinically plausible candidate variables were identified a priori from the literature and routine medical records before model fitting. From these, 12 predictors were selected before multivariable modeling for inclusion in the pre-specified clinical prediction model on the basis of clinical availability within the early perioperative period, redundancy assessment, and intended bedside usability. Exclusion of variables from the final candidate set was based on pre-specified clinical redundancy and usability criteria rather than on statistical significance. No automated stepwise selection procedure was subsequently applied. A supplementary table listing all candidate variables considered before model fitting, their coding, and the reason for inclusion or exclusion is provided (Additional file 2) 0.3,4,10,11 All predictors were routinely available at baseline or during the perioperative period. They were grouped into three domains:

#### Demographic and clinical factors

Age (categorized as < 65, 65–80, > 80 years; dichotomized at > 75 years for the model), sex, BMI (> 30 kg/m²), ASA physical status classification (≥ 3), Garden fracture classification (III/IV), and comorbidities (hypertension, diabetes mellitus, cardiac disease).

#### Laboratory parameters

D-dimer (> 500 ng/mL fibrinogen equivalent units [FEU]), albumin (< 35 g/L), CRP (> 5 mg/L), platelet count (> 400 × 10⁹/L), mean platelet volume (MPV > 10.5 fL), coagulation profile (PT, aPTT, INR). All laboratory samples were collected preoperatively on admission (within 24 h of hospital presentation, before surgical intervention). All laboratory tests were performed in the hospital’s ISO 15,189 accredited central laboratory using standardized procedures with daily internal quality control and monthly external quality assessment.

#### Perioperative factors

Time from injury to surgery (> 48 h), type of anesthesia (regional vs. general), duration of surgery (> 90 min), intraoperative blood loss (> 500 mL), perioperative hypotension (mean arterial pressure < 65 mmHg for > 20 min), blood transfusion requirement (> 2 units), and type of surgical procedure (internal fixation vs. arthroplasty).

### Missing data

Missing data were assessed for each variable. The proportion of missing values was < 2% for all predictors. We used a complete-case approach given the very low rate of missingness, which is considered acceptable when < 5% of data are missing [14]. Sensitivity analyses comparing complete-case results with multiple imputation (5 imputed datasets) confirmed no meaningful differences in point estimates or model performance (imputed AUC 0.81, 95% CI: 0.77–0.85, vs. complete-case AUC 0.81).

### Sample size considerations

Sample size adequacy for model development was assessed in the development cohort, because model fitting was performed in that dataset. The development cohort included 221 DVT events among 1,829 patients. With 12 predictors in the final pre-specified multivariable model, the events-per-variable (EPV) ratio was 18.4 (221/12). In addition, 25 clinically plausible variables were initially screened from the literature and routine clinical records. Before model fitting, 12 predictors were pre-specified for inclusion in the final multivariable model based on clinical availability, redundancy assessment, and intended bedside usability; no data-driven stepwise selection was applied thereafter. When calculated conservatively against the 25 clinically plausible candidate variables considered before model fitting, the EPV was 8.8 (221/25). These values suggest that the final model was reasonably supported for regression-based development, although the more conservative EPV estimate indicates that some optimism remains possible and supports the use of bootstrap internal validation [15]. 

### Statistical analysis

#### Model development

Univariate associations between each candidate predictor and DVT were assessed using chi-square tests, t-tests, or Mann–Whitney U tests as appropriate. The final multivariable logistic regression model included 12 predictors selected before model fitting on clinical and practical grounds. Univariable analyses were performed only to summarize unadjusted associations and were not used to determine predictor inclusion. Multicollinearity was assessed using the variance inflation factor (VIF); all predictors had VIF < 2.5, indicating no concerning collinearity. Regression coefficients were converted into a simplified two-tier integer score. Predictors were assigned 1 or 2 points according to coefficient magnitude, with a pre-set maximum of 2 points per predictor and clinical judgment applied near the tier boundary; no formal nearest-integer rounding algorithm was used.

#### Internal validation

Optimism in model performance was estimated using 1,000 bootstrap resamples of the development cohort. In each resample, the full model development process was repeated — retaining all 12 pre-specified predictors without further selection — and the resulting model was tested on the original dataset to calculate the optimism-corrected AUC (C-statistic). The simplified integer DVTS was also evaluated in each bootstrap resample using the fixed point-allocation algorithm to obtain its optimism-corrected discrimination.

#### Model performance

Discrimination was assessed using the AUC with 95% confidence intervals. Calibration was evaluated using a calibration plot (predicted vs. observed probabilities using LOWESS smoothing), calibration slope, calibration-in-the-large (CITL), the Hosmer–Lemeshow test, and the Brier score [16]. Although we report the Hosmer–Lemeshow statistic, we prioritize the calibration slope and plot as more informative measures of calibration. Clinical utility of the shrinkage-adjusted logistic model was assessed with decision curve analysis (DCA), which plots net benefit against threshold probabilities [17]. Sensitivity, specificity, positive predictive value, and negative predictive value were evaluated at the development-derived exploratory cut-offs of DVTS ≥ 5 and ≥ 9, which were subsequently applied without modification in the temporal validation cohort, not as validated treatment thresholds for current clinical use. The intended prediction time point was the early postoperative window, after surgery and once all perioperative variables were available, before the scheduled day-7 reassessment.

#### Temporal validation

The regression coefficients from the 2018–2023 development model were multiplied by a uniform shrinkage factor (0.92), estimated as the bootstrap-based calibration slope (heuristic shrinkage), and the shrinkage-adjusted model was applied without re-estimation to the 2024 cohort. Discrimination and calibration metrics were recalculated. As secondary exploratory analyses, the Caprini, Padua, and IMPROVE scores were retrospectively calculated for all patients in the temporal validation cohort (*n* = 412) using the same baseline data available in the medical records; each score was computed according to its original published algorithm. Net reclassification improvement (NRI) and integrated discrimination improvement (IDI) were calculated in the validation cohort comparing the shrinkage-adjusted logistic model with the Caprini score. A two-sample bootstrap test of AUC difference (2,000 resamples) was used to compare AUC values between the development and validation cohorts; because the temporal split produced two independent (non-overlapping) samples, no correction for within-subject correlation was required.

#### Inter-rater reliability

No formal multiplicity adjustment was applied to exploratory secondary analyses (comparator scores, subgroup AUC, NRI/IDI, inter-rater reliability); these results should be interpreted cautiously. To assess clinical feasibility, 42 healthcare providers (14 orthopedic residents, 14 orthopedic specialists, and 14 nurses) independently scored a random sample of 50 patient cases using the DVTS. Providers scored cases independently without access to each other’s assessments. Inter-rater agreement was quantified using Fleiss’ kappa with 95% confidence intervals.

All statistical analyses were performed using R version 4.3.2 (R Foundation for Statistical Computing, Vienna, Austria) with packages rms, pROC, CalibrationCurves, and dcurves. Pairwise differences in AUC between the shrinkage-adjusted logistic model and each comparator score, calculated in the same patients, were assessed using DeLong’s test for correlated ROC curves. A two-sided p-value < 0.05 was considered statistically significant.

## Results

### Study population

Of 2,648 patients screened, 2,241 met inclusion criteria. The mean age was 75.3 ± 9.9 years (range: 42–96), with 62.4% female patients. The majority (68.7%) were older than 65 years and 24.7% were older than 80 years. ASA score ≥ 3 was present in 48.4%, and Garden type III–IV fractures constituted 57.7%. The most common comorbidities were hypertension (54.3%), cardiac disease (23.9%), and diabetes mellitus (21.8%). D-dimer levels exceeded 500 ng/mL in 43.2% of patients, and 38.7% had hypoalbuminemia (< 35 g/L). Complete baseline characteristics stratified by DVT status are presented in Table 1.

**Table 1:** Baseline demographic and clinical characteristics stratified by DVT occurrence

Characteristic	Total (*n* = 2,241)	DVT (*n* = 269)	Non-DVT (*n* = 1,972)	*P* value
**Demographics**				
Age, years (mean ± SD)	75.3 ± 9.9	78.6 ± 8.4	74.8 ± 10.1	< 0.001
**Age category**,** n (%)**				0.002
<65 years	702 (31.3)	68 (25.3)	634 (32.2)	
65–80 years	986 (44.0)	112 (41.6)	874 (44.3)	
>80 years	553 (24.7)	89 (33.1)	464 (23.5)	
Female, n (%)	1,398 (62.4)	176 (65.4)	1,222 (62.0)	0.284
BMI, kg/m² (mean ± SD)	26.9 ± 4.0	28.3 ± 4.2	26.7 ± 3.9	0.003
**Clinical Parameters**				
**ASA classification**,** n (%)**				0.002
ASA 1–2	1,156 (51.6)	84 (31.2)	1,072 (54.4)	
ASA 3	814 (36.3)	142 (52.8)	672 (34.1)	
ASA 4	271 (12.1)	43 (16.0)	228 (11.6)	
**Garden classification**,** n (%)**				0.042
Garden I–II	947 (42.3)	98 (36.4)	849 (43.1)	
Garden III–IV	1,294 (57.7)	171 (63.6)	1,123 (56.9)	
**Comorbidities**				
Hypertension, n (%)	1,217 (54.3)	156 (58.0)	1,061 (53.8)	0.205
Diabetes mellitus, n (%)	489 (21.8)	67 (24.9)	422 (21.4)	0.198
Cardiac disease, n (%)	536 (23.9)	78 (29.0)	458 (23.2)	0.038
CKD, n (%)	271 (12.1)	42 (15.6)	229 (11.6)	0.067
**Key Laboratory Parameters**				
D-dimer, ng/mL (mean ± SD)	523 ± 198	862 ± 234	486 ± 178	< 0.001
Albumin, g/L (mean ± SD)	35.8 ± 5.4	31.2 ± 4.8	36.8 ± 5.2	< 0.001
Platelets, ×10⁹/L (mean ± SD)	248 ± 76	287 ± 89	242 ± 73	0.002
CRP, mg/L (median [IQR])	8.4 [4.2–15.6]	12.8 [6.4–22.3]	7.8 [3.9–14.2]	< 0.001

Bold P-values indicate statistical significance (*P* < 0.05). P-values for categorical variables represent chi-square test across all categories. SD, standard deviation; IQR, interquartile range; BMI, body mass index; ASA, American Society of Anesthesiologists; CKD, chronic kidney disease; CRP, C-reactive protein

### DVT incidence and timing

**Fig. 2:**
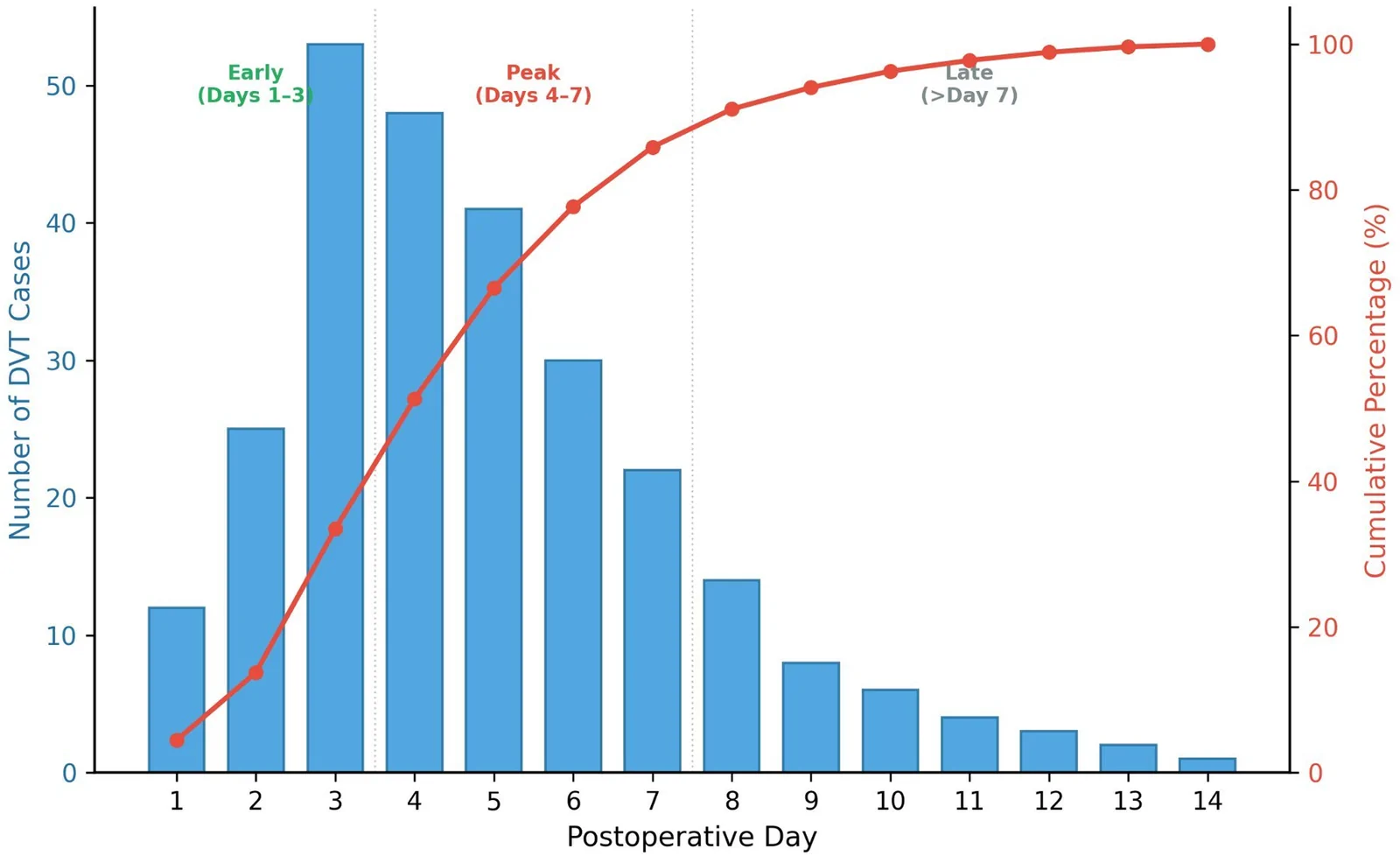
Temporal distribution of the day of DVT diagnosis following femoral neck fracture surgery. The x-axis represents postoperative days, and the y-axis shows the number and cumulative percentage of DVT diagnoses

DVT occurred in 269 patients, yielding an overall incidence of 12.0% (95% CI: 10.7–13.4%). The median time to DVT diagnosis was 5 days (IQR: 3–8 days), with the highest combined frequency observed during postoperative days 4–5 (33.1% of cases). Early DVT (Days 1–3 postoperatively) occurred in 90 patients (33.5%), peak-period DVT (Days 4–7) in 134 patients (49.8%), and late DVT (> Day 7) in 45 patients (16.7%). Proximal DVT was diagnosed in 174 cases (64.7%), distal DVT in 95 cases (35.3%), and bilateral involvement in 30 cases (11.2%). The operated limb was affected in 82.3% of cases. The temporal distribution of DVT is illustrated in Fig. 2.

### Unadjusted and adjusted associations of prespecified predictors

Comparison of laboratory parameters between DVT and non-DVT groups is shown in Table 2. D-dimer levels were markedly elevated in the DVT group (862 ± 234 ng/mL vs. 486 ± 178 ng/mL, *p* < 0.001), as were platelet counts (287 ± 89 vs. 242 ± 73 × 10^9^/L, *p* = 0.002), MPV (10.8 ± 1.2 vs. 10.1 ± 1.0 fL, *p* = 0.004), and CRP levels (median 12.8 vs. 7.8 mg/L, *p* < 0.001). Albumin levels were significantly lower in the DVT group (31.2 ± 4.8 vs. 36.8 ± 5.2 g/L, *p* < 0.001).

**Table 2:** Comparison of laboratory parameters between DVT and non-DVT groups

Parameter	Total (*n* = 2,241)	DVT (*n* = 269)	Non-DVT (*n* = 1,972)	*P* value
**Coagulation Markers**				
D-dimer, ng/mL (mean ± SD)	523 ± 198	862 ± 234	486 ± 178	< 0.001
D-dimer > 500 ng/mL, n (%)	968 (43.2)	198 (73.6)	770 (39.0)	< 0.001
PT, seconds (mean ± SD)	12.8 ± 1.4	13.1 ± 1.6	12.7 ± 1.3	0.092
aPTT, seconds (mean ± SD)	28.4 ± 3.8	29.2 ± 4.1	28.3 ± 3.7	0.134
INR (mean ± SD)	1.05 ± 0.12	1.07 ± 0.14	1.05 ± 0.11	0.218
Fibrinogen, g/L (mean ± SD)	3.8 ± 1.1	4.3 ± 1.3	3.7 ± 1.0	0.002
**Platelet Indices**				
Platelets, ×10⁹/L (mean ± SD)	248 ± 76	287 ± 89	242 ± 73	0.002
Platelets > 400 × 10⁹/L, n (%)	178 (7.9)	34 (12.6)	144 (7.3)	0.003
MPV, fL (mean ± SD)	10.2 ± 1.1	10.8 ± 1.2	10.1 ± 1.0	0.004
PDW, % (mean ± SD)	13.4 ± 2.8	14.2 ± 3.1	13.3 ± 2.7	0.038
**Inflammatory and Nutritional Markers**				
CRP, mg/L (median [IQR])	8.4 [4.2–15.6]	12.8 [6.4–22.3]	7.8 [3.9–14.2]	< 0.001
CRP > 5 mg/L, n (%)	1,482 (66.1)	214 (79.6)	1,268 (64.3)	< 0.001
Albumin, g/L (mean ± SD)	35.8 ± 5.4	31.2 ± 4.8	36.8 ± 5.2	< 0.001
Albumin < 35 g/L, n (%)	867 (38.7)	178 (66.2)	689 (34.9)	< 0.001
WBC, ×10⁹/L (mean ± SD)	9.2 ± 3.4	10.1 ± 3.8	9.1 ± 3.3	0.046
Hemoglobin, g/L (mean ± SD)	118 ± 18	112 ± 19	119 ± 17	0.008

Continuous variables: mean ± SD (independent t-test) or median [IQR] (Mann–Whitney U). Categorical variables: n (%) (chi-square). SD, standard deviation; IQR, interquartile range; PT, prothrombin time; aPTT, activated partial thromboplastin time; INR, international normalized ratio; MPV, mean platelet volume; PDW, platelet distribution width; CRP, C-reactive protein; WBC, white blood cell count

**Fig. 3:**
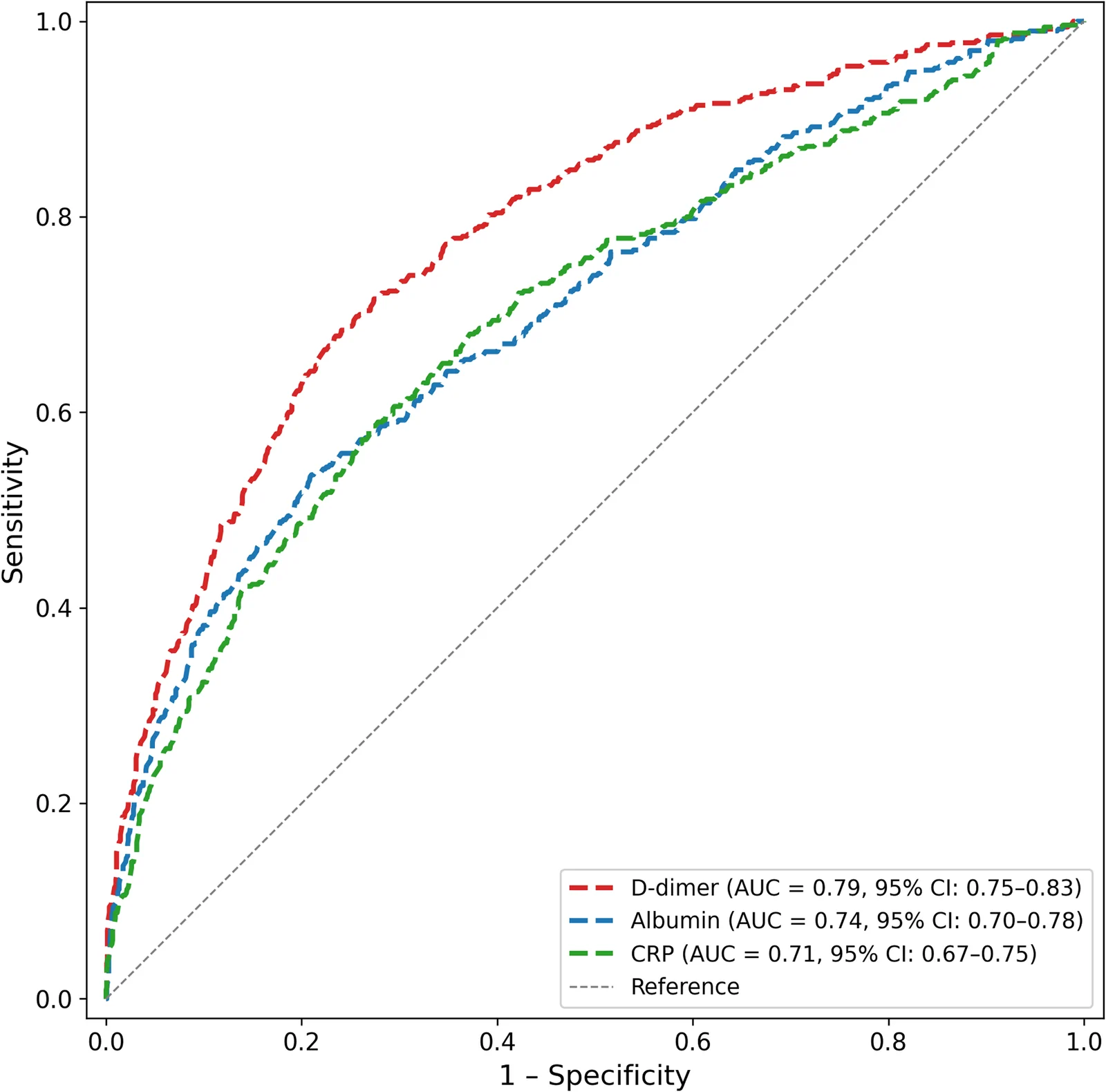
Receiver operating characteristic (ROC) curves for individual laboratory parameters in DVT prediction. AUC values with 95% confidence intervals are shown for D-dimer, albumin, and CRP in the development cohort (*n* = 1,829). Performance of the full logistic model and the simplified DVTS score is reported separately in Table 6

ROC curve analysis for individual laboratory parameters revealed AUC values of 0.79 (95% CI: 0.75–0.83) for D-dimer, 0.74 (95% CI: 0.70–0.78) for albumin, and 0.71 (95% CI: 0.67–0.75) for CRP (Fig. 3). Figure 3 displays ROC curves for the individual laboratory parameters only. Performance of the full logistic model and the simplified integer-based DVTS is reported separately in Table 6 to avoid conflating apparent model performance with that of the bedside score.

Table 3. Multivariable regression coefficients for the prespecified predictors.

**Table 3:** Multivariable regression coefficients for the prespecified predictors

Risk Factor	β-coefficient	Adjusted OR	95% CI	*P* value	Points
**Demographic and Clinical Factors**					
Age > 75 years	0.831	2.30	1.80–2.94	< 0.001	2
BMI > 30 kg/m²	0.458	1.58	1.18–2.12	0.003	1
ASA score ≥ 3	0.591	1.81	1.41–2.32	< 0.001	2
Garden III/IV	0.473	1.60	1.21–2.13	0.003	1
**Laboratory Parameters**					
D-dimer > 500 ng/mL	1.028	2.80	2.11–3.72	< 0.001	2
Albumin < 35 g/L	0.644	1.90	1.49–2.43	< 0.001	2
CRP > 5 mg/L	0.584	1.79	1.38–2.32	< 0.001	1
Platelet count > 400 × 10⁹/L	0.408	1.50	1.12–2.02	0.008	1
**Perioperative Factors**					
Time to surgery > 48 h	0.528	1.70	1.30–2.21	0.002	1
Operation > 90 min	0.461	1.59	1.20–2.10	0.004	1
Perioperative hypotension†	0.536	1.71	1.31–2.24	0.002	1
Blood transfusion > 2 units	0.416	1.52	1.13–2.04	0.007	1

†Defined as MAP < 65 mmHg for > 20 min. OR, odds ratio; CI, confidence interval; MAP, mean arterial pressure. β-coefficients and OR/CI in this table are original (pre-shrinkage) estimates. The regression equation in the text uses shrinkage-adjusted coefficients (uniform shrinkage factor 0.92); original and shrinkage-adjusted model performances are compared in Additional file 3. Predictors with larger coefficients received 2 points; remaining predictors received 1 point each. Total score range: 0–16 points

### DVT Risk Score (DVTS) development

**Fig. 4:**
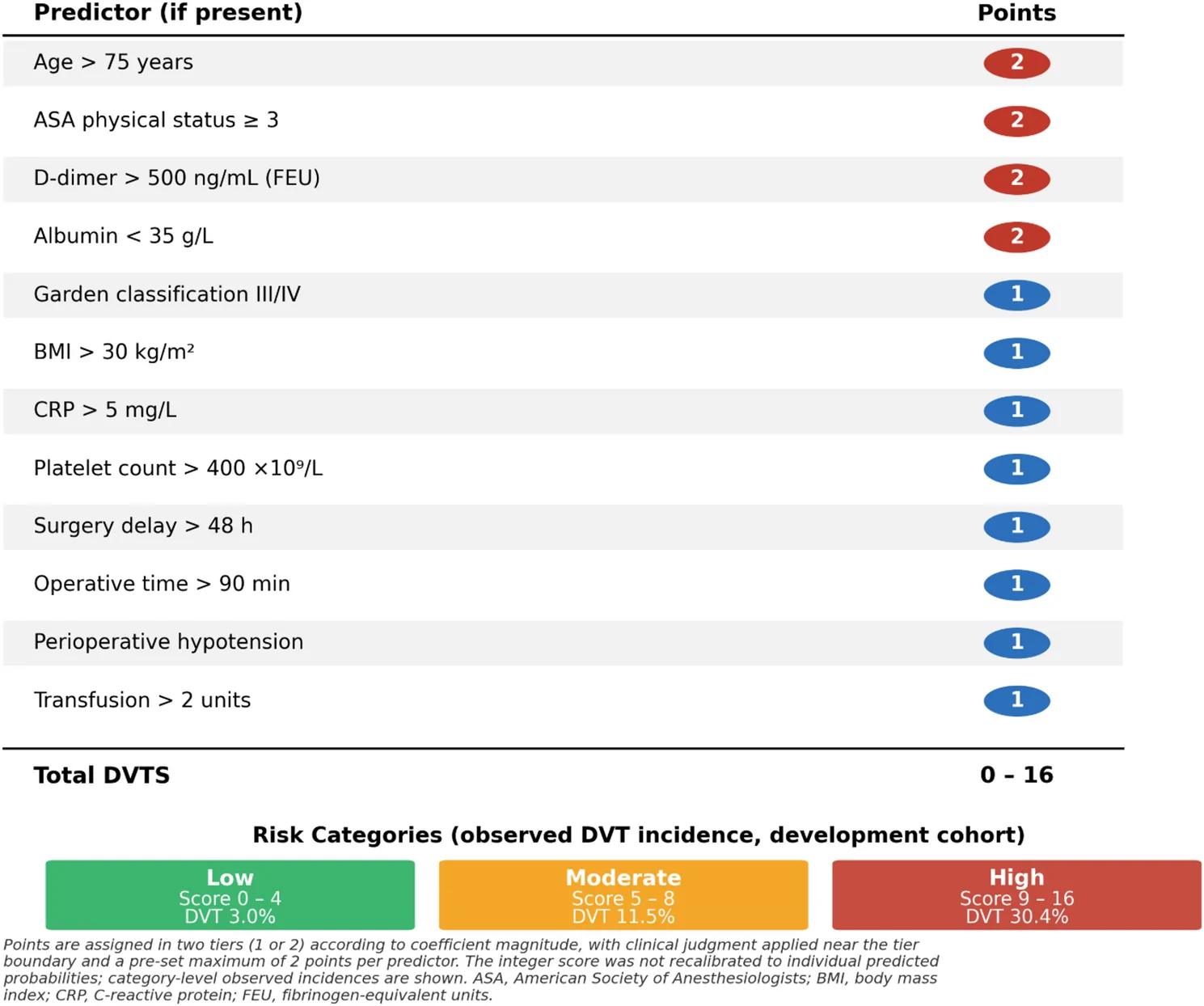
DVT Risk Score (DVTS) scoring chart. Each predictor present at the early postoperative assessment contributes 1 or 2 points (two-tier assignment, maximum 2 points per predictor), giving a total score of 0–16. The three risk categories (low, 0–4; moderate, 5–8; high, 9–16) are shown with their observed DVT incidence in the development cohort. The simplified integer score was not recalibrated to individual predicted probabilities; category-level observed incidences are presented rather than a continuous predicted-probability scale. ASA, American Society of Anesthesiologists; BMI, body mass index; CRP, C-reactive protein

Based on the regression coefficients, a weighted integer-based scoring system was constructed. Point weights were assigned by ranking the shrinkage-adjusted coefficients and grouping them into two tiers, with a pre-set maximum of 2 points per predictor to preserve bedside simplicity. The four predictors in the upper tier (age > 75 years, D-dimer > 500 ng/mL, albumin < 35 g/L, and ASA score ≥ 3) received 2 points each, and the remaining eight received 1 point each. Because several coefficients were numerically close near the tier boundary (for example, ASA ≥ 3 [β = 0.591] and CRP > 5 mg/L [β = 0.584]), the final tier assignment incorporated clinical judgment in addition to coefficient magnitude; ASA class was placed in the upper tier given its established, guideline-level role in perioperative risk assessment. We did not re-estimate the intercept or recalibrate the simplified integer score separately from the shrinkage-adjusted logistic model, and the two are compared directly in Additional file 3. Because simplification from a regression model to an integer score necessarily introduces some subjectivity and information loss, the full shrinkage-adjusted regression equation is also reported to preserve transparency (see below). The total score ranges from 0 to 16 points. Risk-category thresholds were derived in the development cohort based on the distribution of predicted probabilities (targeting approximate DVT risk levels of < 5%, 5–20%, and > 20%) and were subsequently evaluated, without modification, in the temporal validation cohort. This yielded three categories: low (0–4 points), moderate (5–8 points), and high risk (9–16 points). These should be regarded as exploratory classification thresholds derived from the development data rather than validated clinical decision thresholds. The complete scoring system is presented in Table 4. For full model transparency (TRIPOD item 15a), the logistic regression equation is: P(DVT) = 1 / (1 + exp[−(− 3.21 + 0.765×Age > 75 + 0.946×Ddimer > 500 + 0.593×Albumin < 35 + 0.544×ASA ≥ 3 + 0.435×GardenIII-IV + 0.421×BMI > 30 + 0.537×CRP > 5 + 0.375×Platelet > 400 + 0.486×SurgDelay>48 h + 0.424×OpTime>90 min + 0.493×Hypotension + 0.383×Transfusion > 2)]), where all predictors are binary (1 = present, 0 = absent) and coefficients are shrinkage-adjusted (uniform shrinkage factor 0.92). The intercept (− 3.21) was re-estimated after applying the uniform shrinkage factor to the predictor coefficients to maintain calibration-in-the-large. The corresponding scoring chart is presented in Fig. 4.

**Table 4:** DVT Risk Score (DVTS) — Components and Point Allocation

Predictor	Threshold	Points	Data Source
**Demographic and Clinical (max 6 points)**			
Age	> 75 years	2	Medical record
ASA physical status	≥ 3	2	Anesthesia assessment
Garden classification	III or IV	1	Radiographic report
Body mass index	> 30 kg/m²	1	Admission data
**Laboratory Parameters (max 6 points)**			
D-dimer	> 500 ng/mL	2	Preoperative labs
Albumin	< 35 g/L	2	Preoperative labs
C-reactive protein	> 5 mg/L	1	Preoperative labs
Platelet count	> 400 × 10⁹/L	1	Preoperative labs
**Perioperative Factors (max 4 points)**			
Time to surgery	> 48 h	1	Operative log
Duration of surgery	> 90 min	1	Operative log
Perioperative hypotension†	MAP < 65 mmHg, > 20 min	1	Anesthesia record
Blood transfusion	> 2 units	1	Operative/ward record
**Total Score Range: 0–16 points**			
**Risk Categories**:			
Low risk	0–4 points		DVT incidence: 3.3%
Moderate risk	5–8 points		DVT incidence: 11.7%
High risk	9–16 points		DVT incidence: 29.2%

†MAP, mean arterial pressure. Points were assigned in two tiers (1 or 2 points) according to coefficient magnitude, with a pre-set maximum of 2 points per predictor and clinical judgment applied near the tier boundary; they are not a direct proportional transformation of the β-coefficients. Risk categories defined by score thresholds at clinically meaningful DVT probability levels (≈ 5% and ≈ 20%). All predictors are routinely available by the early postoperative assessment window

In clinical practice, the DVTS is calculated by summing the points for each predictor present at the time of assessment (all variables available by the early postoperative assessment window). For example, a 78-year-old patient (2 points) with ASA 3 (2 points), D-dimer 800 ng/mL (2 points), albumin 32 g/L (2 points), and surgery delay > 48 h (1 point) would receive a total DVTS of 9, classifying them as high risk. Because the simplified integer score was not separately recalibrated to predicted probabilities, we report the risk category rather than an individual probability for a given total score. Patients scoring ≥ 5 (moderate-to-high risk) may represent a candidate higher-risk group for future external validation studies and prospective research focused on clinically relevant thromboembolic outcomes; this threshold should not be interpreted as an indication for intensified anticoagulation, whereas patients scoring 0–4 represent a lower-risk group under the current classification.

### Model performance

**Fig. 5:**
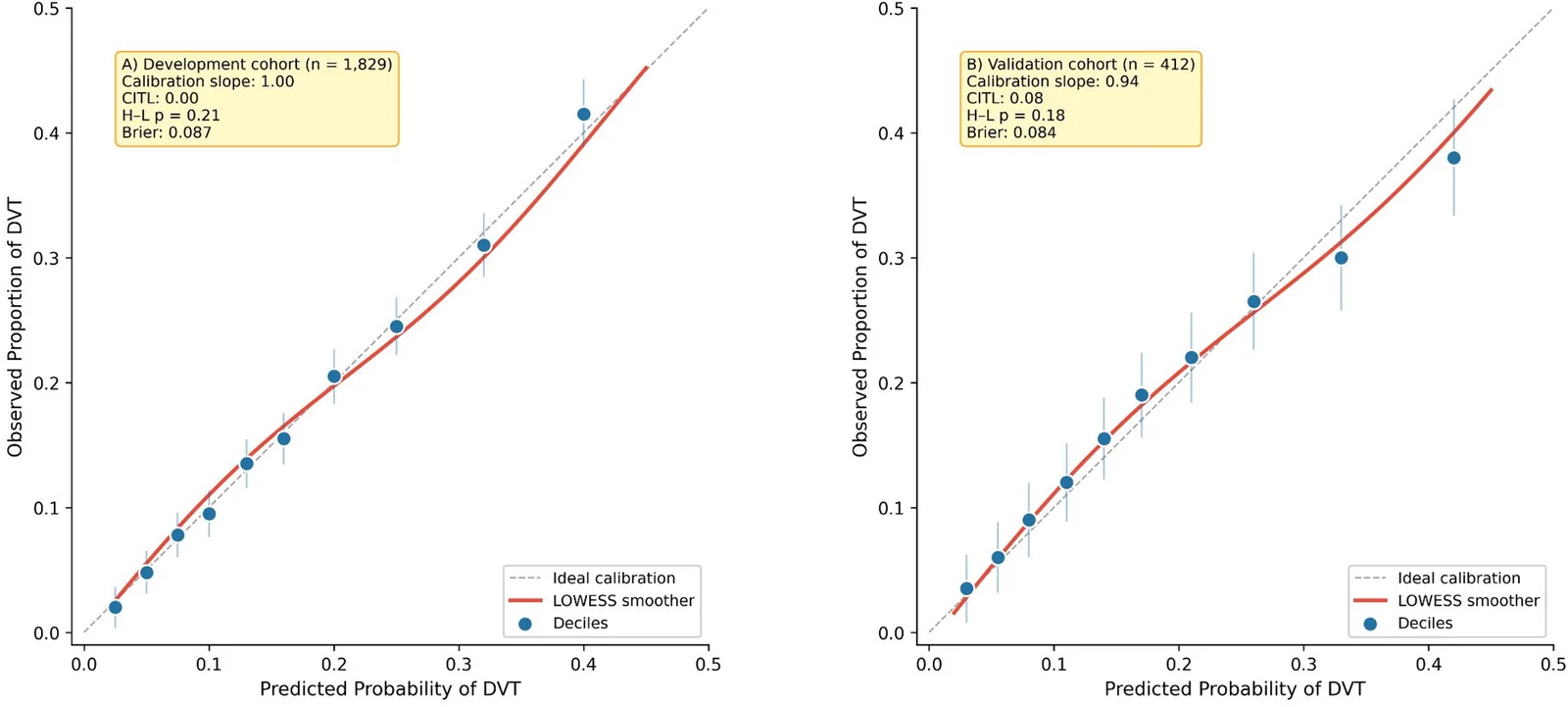
(Shrinkage-adjusted logistic model.) Calibration plots for the DVTS. Panel **A**: development cohort (*n* = 1,829); Panel **B**: temporal validation cohort (*n* = 412). Circles represent observed DVT proportions within predicted probability deciles; vertical bars indicate 95% confidence intervals. The LOWESS-smoothed calibration curve (red line) shows the continuous relationship between predicted and observed probabilities. The diagonal dashed line represents perfect calibration (slope = 1.00). Annotated metrics: calibration slope, calibration-in-the-large (CITL), Hosmer–Lemeshow p-value, and Brier score. Temporal validation cohort calibration (slope 0.94) is reported in the text and Table 6

In the development cohort, the simplified integer DVTS demonstrated an apparent AUC of 0.81 (95% CI: 0.77–0.85); the underlying logistic model had an apparent AUC of 0.83 (95% CI: 0.79–0.87). Because multiplying all coefficients by a uniform shrinkage factor preserves the rank order of the linear predictor, the shrinkage-adjusted and unshrunk logistic models share the same apparent discrimination (AUC 0.83). Bootstrap internal validation yielded optimism-corrected AUCs of 0.80 (95% CI: 0.76–0.84) for the shrinkage-adjusted logistic model and 0.79 (95% CI: 0.75–0.83) for the simplified integer DVTS (optimism = 0.01 and 0.02, respectively). For the shrinkage-adjusted logistic model, development calibration was good, with a slope of 1.00, CITL of 0.00, Hosmer–Lemeshow *p* = 0.21, and a Brier score of 0.087 (95% CI: 0.074–0.100) (Fig. 5).

**Fig. 6:**
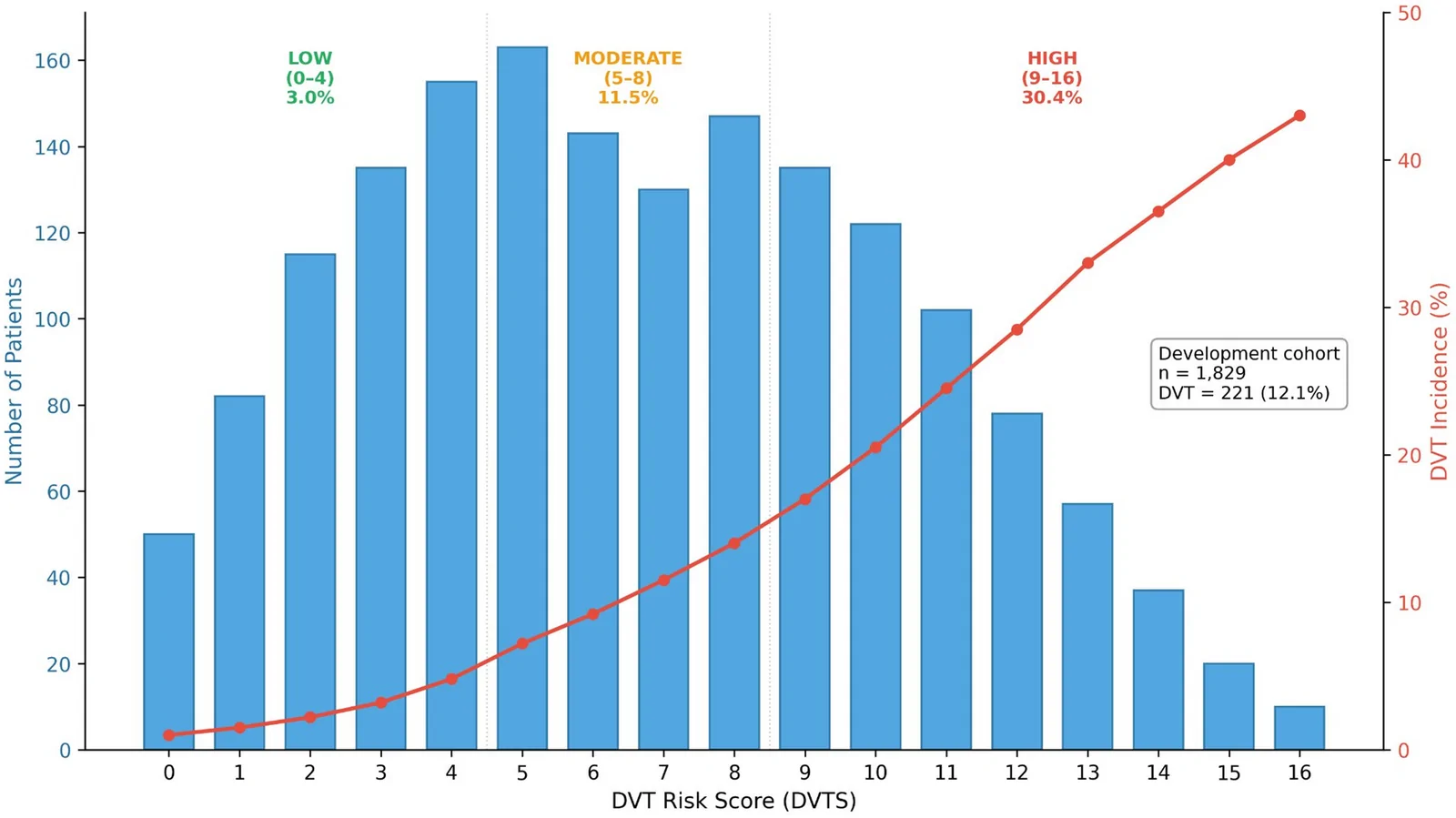
Risk score distribution and corresponding DVT incidence by score value. Bars represent the number of patients at each score value (left y-axis), and the line represents the observed DVT incidence (right y-axis). Risk categories (low, moderate, high) are indicated by colored background zones. Score distribution and risk category incidence rates shown for the development cohort only (*n* = 1,829); category-specific incidences therefore differ slightly from the combined-cohort values reported in Table 5. Risk category thresholds were defined in the development cohort

When the DVTS was applied to the full study cohort (*n* = 2,241; development and validation combined) as a post-hoc descriptive analysis, DVT incidence increased progressively across risk categories: 3.3% (29/892) in the low-risk group, 11.7% (102/876) in the moderate-risk group, and 29.2% (138/473) in the high-risk group. In the development cohort alone (*n* = 1,829), corresponding DVT incidences were 3.0% (22/733), 11.5% (82/711), and 30.4% (117/385), respectively; in the temporal validation cohort (*n* = 412), incidences were 4.4% (7/159), 12.1% (20/165), and 23.9% (21/88) (*p* < 0.001 for trend). The relative risk for DVT in the high-risk group compared with the low-risk group was 9.1 (95% CI: 6.3–13.2). Detailed performance by risk category is presented in Table 5, and the score distribution with corresponding DVT rates in Fig. 6.

**Table 5:** DVT incidence and relative risk across DVTS risk categories

Risk Category	Score	Patients, *n* (%)	DVT, *n*	Incidence (%)	RR (95% CI)
Low Risk					
Total	0–4	892 (39.8)	29	3.3	Reference
ASA 1–2	–	624 (27.8)	16	2.6	0.8 (0.5–1.3)
ASA ≥ 3	–	268 (12.0)	13	4.8	1.5 (1.1–2.0)
**Moderate Risk**					
Total	5–8	876 (39.1)	102	11.7	3.6 (2.4–5.4)
ASA 1–2	–	384 (17.1)	38	9.9	3.1 (1.9–4.8)
ASA ≥ 3	–	492 (22.0)	64	13.0	4.0 (2.7–6.1)
**High Risk**					
Total	9–16	473 (21.1)	138	29.2	9.1 (6.3–13.2)
ASA 1–2	–	148 (6.6)	30	20.3	6.3 (4.0–9.9)
ASA ≥ 3	–	325 (14.5)	108	33.2	10.3 (7.1–15.1)
**Overall**					
Total	0–16	2,241 (100)	269	12.0	–

*P* < 0.001 for trend across risk categories. Incidences are based on the full study cohort (*n* = 2,241; development and validation combined). Development-cohort-specific incidences are shown in Fig. 6. RR, relative risk; CI, confidence interval; ASA, American Society of Anesthesiologists

**Fig. 7:**
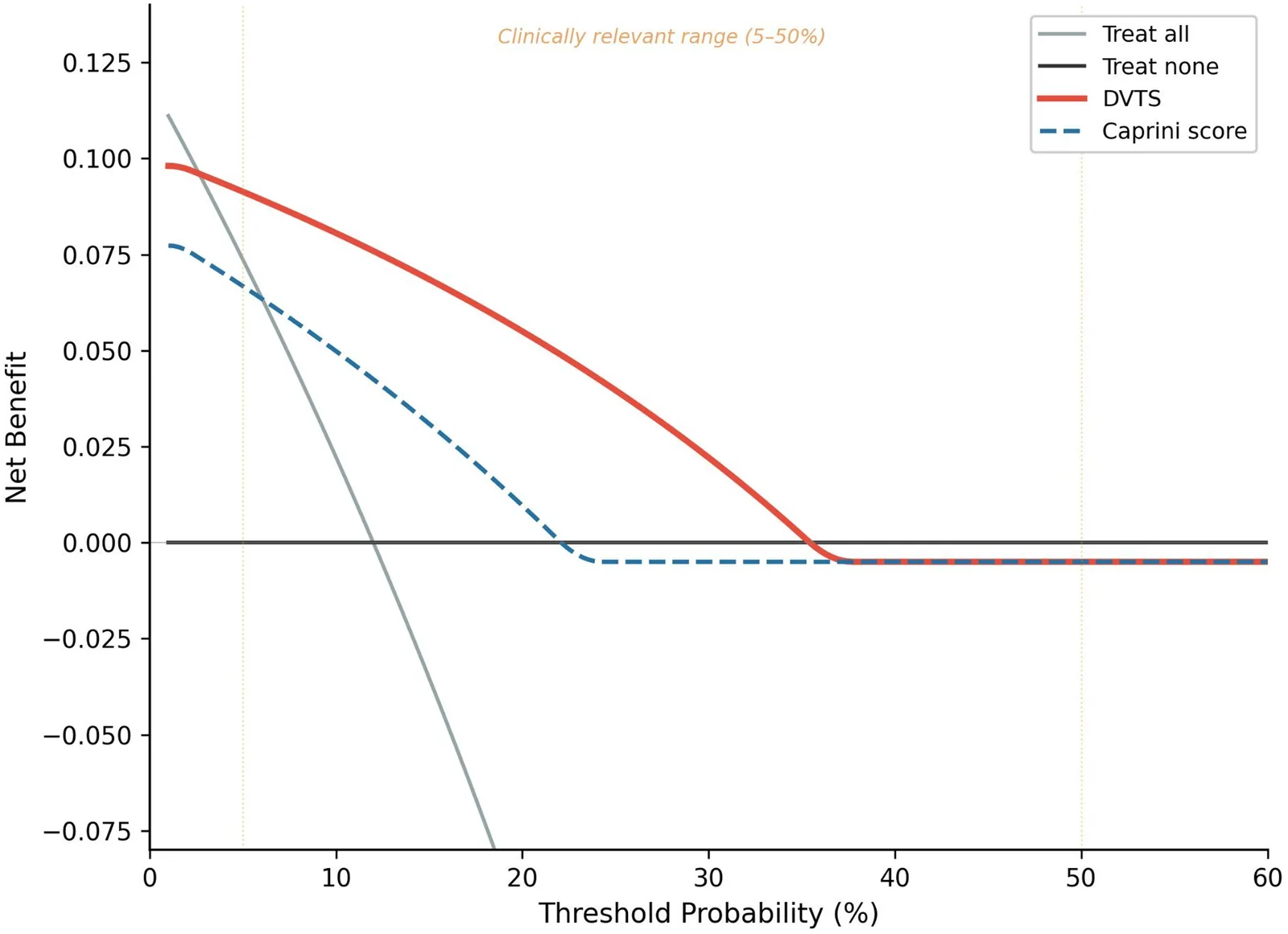
(Based on the shrinkage-adjusted logistic model.) Decision curve analysis comparing the shrinkage-adjusted logistic model with the Caprini score, treat-all, and treat-none strategies. The y-axis shows net benefit, and the x-axis shows threshold probability (%). The shrinkage-adjusted logistic model (red solid line) shows higher estimated net benefit than the Caprini score across threshold probabilities of 5%–50% in the temporal validation cohort. These curves represent model-based decision estimates and not observed treatment effects. Analysis performed in the temporal validation cohort (*n* = 412). DCA, decision curve analysis; DVTS, DVT Risk Score. Because both prediction approaches were evaluated in the same 412 patients, their net-benefit curves are correlated, and the higher estimated net benefit of the shrinkage-adjusted logistic model should be interpreted cautiously. Notably, the Caprini score net benefit falls below zero at threshold probabilities above approximately 22%, suggesting diminishing utility of the Caprini score at higher thresholds in this population

In the temporal validation cohort, decision curve analysis — presented only as an exploratory, model-based characterization of net benefit and not as a recommendation to escalate anticoagulation — suggested that the shrinkage-adjusted logistic model may provide greater net benefit than the “treat-all,” “treat-none,” and Caprini-based strategies across threshold probabilities of 5%–50% in this validation cohort. These findings represent model-based estimates of potential decision utility and should not be interpreted as evidence that model-guided prophylaxis improves patient outcomes (Fig. 7). At a threshold probability of 10%, chosen as a clinically plausible exploratory decision threshold, the shrinkage-adjusted logistic model had a net benefit of 0.062, compared with 0.021 for the Caprini score. At our institution’s DVT incidence of 12%, these model-based estimates should not be interpreted as observed treatment effects and require prospective validation in an impact study. Because both prediction approaches were evaluated in the same 412 patients, their net-benefit curves are correlated, and the higher estimated net benefit of the shrinkage-adjusted logistic model should be interpreted accordingly. Notably, the Caprini score net benefit falls below zero at threshold probabilities above approximately 22%, suggesting diminishing utility of the Caprini score at higher thresholds in this population.

### Temporal validation

In the temporal validation cohort (*n* = 412), the shrinkage-adjusted logistic model maintained good discrimination, with an AUC of 0.82 (95% CI: 0.77–0.87), comparable to the development cohort (absolute difference 0.01; *p* = 0.67 by two-sample bootstrap test). The simplified integer DVTS retained good discrimination in the temporal validation cohort (AUC 0.81, 95% CI: 0.76–0.86). Calibration slope was 0.94 (95% CI: 0.89–0.99) and CITL of 0.08 (95% CI: −0.12 to 0.28), indicating slight over-estimation of predicted probabilities at higher risk levels; however, calibration remained acceptable overall in this single-center temporal validation cohort. For the simplified integer DVTS, the development-derived cut-off of ≥ 5 in the temporal validation cohort yielded a sensitivity of 85.4% and specificity of 41.8%, with a negative predictive value of 95.6% and positive predictive value of 16.2%; a DVTS < 5 was associated with a negative predictive value of 95.6% in this cohort; however, this finding should not be interpreted as a validated rule-out strategy or as justification for omitting clinically indicated imaging, as DVT was still detected in 7 of 159 (4.4%) low-score patients in the validation cohort. A higher cut-off (DVTS ≥ 9) yielded higher specificity (81.6%) but lower sensitivity (43.8%). These thresholds are exploratory classification cut-offs rather than validated clinical decision thresholds. Complete validation metrics are presented in Table 6.

**Table 6:** Model performance: development, internal validation, and temporal validation

Metric	Shrinkage-adjusted logistic model	Simplified integer DVTS
Development AUC (95% CI)†	0.83 (0.79–0.87)	0.81 (0.77–0.85)
Optimism-corrected AUC (95% CI)	0.80 (0.76–0.84)	0.79 (0.75–0.83)
Uniform shrinkage factor	0.92	—
Temporal-validation AUC (95% CI)	0.82 (0.77–0.87)	0.81 (0.76–0.86)
Calibration slope (validation)	0.94 (0.89–0.99)	Not separately estimated
CITL (validation)	0.08 (− 0.12 to 0.28)	Not separately estimated
Brier score (validation)	0.084 (0.068–0.100)	Not separately estimated
Hosmer–Lemeshow χ² (validation)	11.0 (df = 8, *p* = 0.18)	—
Sensitivity / specificity at ≥ 5	—	85.4% / 41.8%
Sensitivity / specificity at ≥ 9	—	43.8% / 81.6%
NPV / PPV at ≥ 5	—	95.6% / 16.2%
Decision curve analysis	Performed	Not separately evaluated
NRI / IDI vs. Caprini (validation)	0.38 / 0.11	—

Table 6. Performance metrics are explicitly attributed to either the shrinkage-adjusted logistic model or the simplified integer DVTS. Calibration slope, calibration-in-the-large (CITL), Brier score, and decision curve analysis refer to the shrinkage-adjusted logistic model; threshold-based sensitivity, specificity, NPV, and PPV refer to the simplified integer DVTS. The uniform shrinkage factor (0.92) was estimated as the bootstrap-based calibration slope. †Because a uniform shrinkage factor preserves the rank order of the linear predictor, the shrinkage-adjusted and unshrunk logistic models share the same apparent AUC (0.83). The exploratory cut-offs DVTS ≥5 and ≥9 were derived in the development cohort and applied unchanged in temporal validation; they are not validated decision thresholds. AUC, area under the curve; CI, confidence interval; NRI, net reclassification improvement (category-free); IDI, integrated discrimination improvement; NPV, negative predictive value; PPV, positive predictive value

### Subgroup analyses

**Fig. 8:**
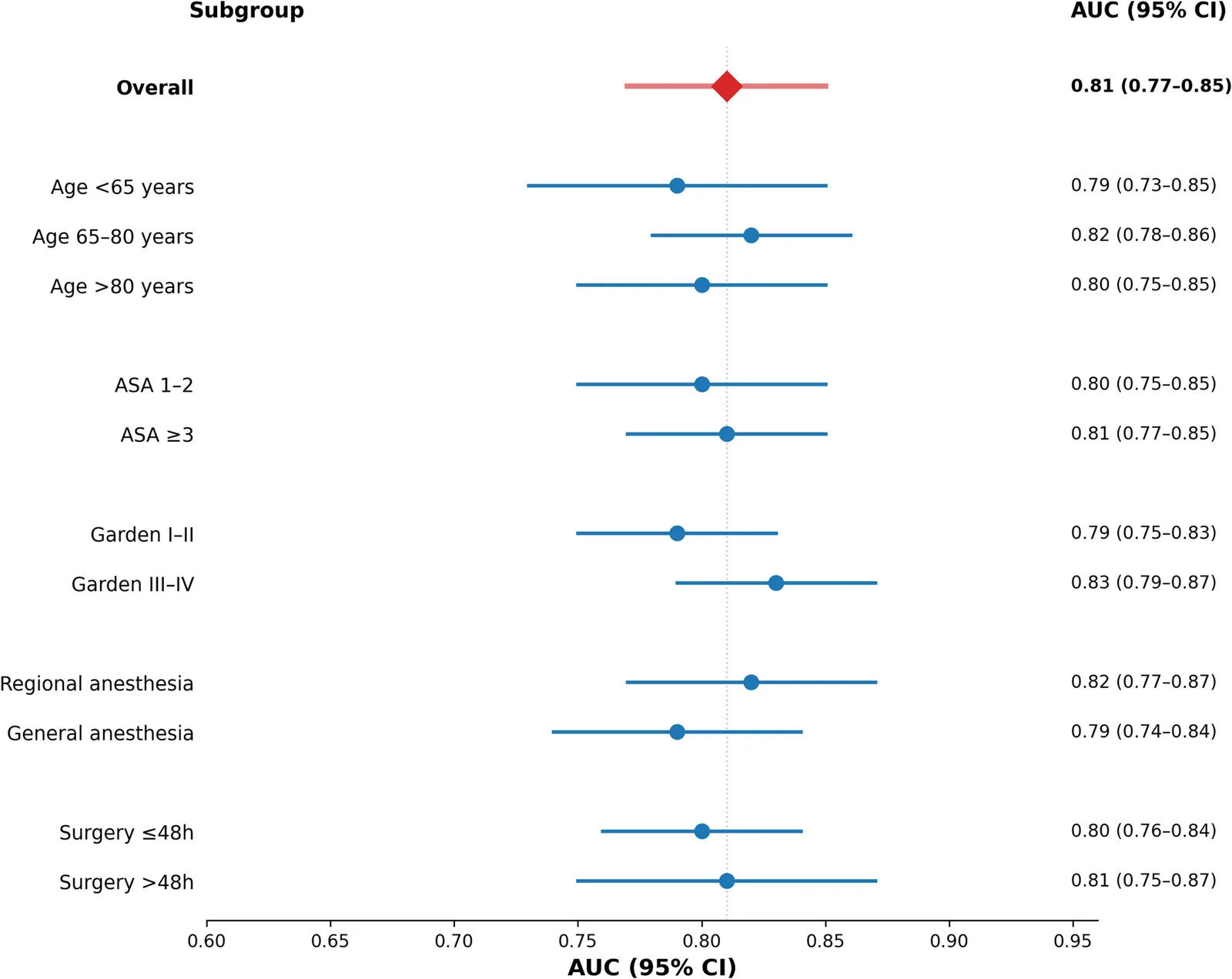
Forest plot of model discrimination (AUC with 95% CI) across predefined subgroups: age categories (< 65, 65–80, > 80 years), ASA classification (1–2 vs. ≥3), Garden classification (I–II vs. III–IV), and anesthesia type (regional vs. general). Subgroup analysis performed in the development cohort (*n* = 1,829). The vertical dashed line represents the overall development AUC. Subgroup analyses are exploratory and underpowered for formal inference. No formal interaction testing was performed, and no global heterogeneity p-value is presented

In exploratory subgroup analyses, the DVTS showed broadly similar discrimination across age, ASA, and fracture-classification strata. Age-stratified AUC values were 0.79 (95% CI: 0.73–0.85) for patients < 65 years, 0.82 (95% CI: 0.78–0.86) for 65–80 years, and 0.80 (95% CI: 0.75–0.85) for > 80 years. Model discrimination was maintained across ASA categories (AUC 0.80 for ASA 1–2 vs. 0.81 for ASA ≥ 3) and Garden classification types (AUC range 0.79–0.83). Subgroup performance is illustrated in Fig. 8.

### Comparison with existing scores

In the temporal validation cohort, the shrinkage-adjusted logistic model showed higher discrimination than the retrospectively calculated Caprini score (AUC 0.82 vs. 0.67, *p* < 0.001), Padua score (AUC 0.82 vs. 0.69, *p* < 0.001), and IMPROVE score (AUC 0.82 vs. 0.71, *p* < 0.001). The category-free (continuous) net reclassification improvement (NRI) for the shrinkage-adjusted logistic model compared with the Caprini score was 0.38 (95% CI: 0.31–0.45, *p* < 0.001; event NRI 0.22, non-event NRI 0.16), and the integrated discrimination improvement (IDI) was 0.11 (95% CI: 0.08–0.14, *p* < 0.001). These comparisons should be interpreted as secondary exploratory analyses within the temporal validation cohort.

### Clinical feasibility

Inter-rater reliability testing among 42 healthcare providers (exploratory reproducibility assessment) who independently scored 50 cases demonstrated substantial agreement (Fleiss κ = 0.87 for the three-tier risk-category classification; classified as ‘almost perfect’ agreement per Landis and Koch 25, 95% CI: 0.83–0.91). There was no significant difference in agreement between physicians and nurses (*p* = 0.34). The mean time required for manual score calculation was 2.^8^±0.^9^ minutes; electronic implementation through the hospital information system reduced this to 1.^6^±0.^4^ minutes (*p* < 0.001).

## Discussion

The shrinkage-adjusted logistic model showed comparable discrimination in the development and temporal validation cohorts (apparent development AUC 0.83; temporal-validation AUC 0.82), with acceptable calibration in temporal validation (slope 0.94). The simplified integer DVTS had an apparent AUC of 0.81 in development and 0.81 (95% CI: 0.76–0.86) in temporal validation. The optimism-corrected AUC in development was 0.80, suggesting limited internal optimism, although some overfitting remains possible given the single-center retrospective design and score simplification. In the development cohort, the final model was supported by 221 events for 12 predictors (EPV 18.4). Decision curve analysis suggested potential decision utility, but this should not be interpreted as proof of clinical benefit. The shrinkage-adjusted logistic model showed higher apparent discrimination than the retrospectively reconstructed comparator scores within this single-center temporal validation cohort.

The observed DVT incidence of 12.0% is consistent with recent literature. A systematic review by Kobayashi et al. reported preoperative DVT rates of 6–62% in hip fracture patients depending on timing and screening method, with most contemporary studies using routine ultrasonography reporting rates of 10–15%.^3^ Cong et al. found a DVT prevalence of 44.^7^% before surgery in hip fracture patients when screening was performed daily, highlighting the importance of standardized screening protocols [10]. Our protocol of screening at 24–48 h and 7 days postoperatively captured a substantial proportion of postoperatively detected events under the institutional surveillance strategy while remaining feasible in routine practice.

Three predictor domains contributed independently to the DVTS: laboratory biomarkers (D-dimer, albumin, CRP, platelets), perioperative factors (surgical delay, operative time, hypotension, transfusion), and baseline demographics/comorbidity (age, ASA, BMI, Garden classification). D-dimer emerged as the strongest single predictor (OR 2.8), which is supported by its established role as a biomarker of fibrin turnover and coagulation activation [4, 18]. The strong predictive value of preoperative hypoalbuminemia (OR 1.9) reflects its role as a marker of malnutrition and systemic inflammation, both of which are established risk factors for thrombosis in elderly surgical patients [19]. Our finding that delayed surgery > 48 h independently predicts DVT (OR 1.7) is consistent with Smith et al., who demonstrated that each day of surgical delay increases DVT risk in hip fracture patients [20]. 

The shrinkage-adjusted logistic model demonstrated higher discrimination in this cohort than the evaluated general-purpose scores when applied to our population. The Caprini score, while validated across surgical specialties, yielded an AUC of only 0.62 in the study by Qiao et al. involving 3,807 arthroplasty patients [7]. Similarly, Xiang et al. reported that a population-specific scoring chart for intertrochanteric fractures achieved an AUC of 0.79, comparable to our model but in a different fracture type and population [9]. Lin et al. similarly developed a scoring chart for lower extremity fractures with comparable methodology [21]. One possible explanation is that DVTS incorporates perioperative and laboratory variables not captured by the compared generic scores; however, this interpretation remains cohort-dependent and requires external validation.

Several important caveats apply to these comparisons. First, the Caprini, Padua, and IMPROVE scores were developed to predict clinically overt, symptomatic VTE, whereas our outcome was predominantly surveillance-detected DVT; the scores were therefore applied to a different outcome than the one for which they were derived. Second, the Padua and IMPROVE scores were designed for acutely ill medical inpatients rather than surgical or orthopedic trauma populations, which may partly explain their lower discrimination here. Third, these analyses represent external validation for the comparator scores but internal (temporal) validation for the DVTS, which confers a methodological advantage to our model; notwithstanding this, the comparator scores retained modest discrimination, with AUC values ranging from 0.67 to 0.71 in this cohort. Fourth, orthopedic- and fracture-specific DVT prediction models have been reported in other populations, including several derived from Chinese patient cohorts; these were not re-derived or recalibrated in our data because they use partly different predictor sets and assay-dependent thresholds, and head-to-head recalibration was beyond the scope of this single-center study. These comparisons should therefore be regarded as exploratory and hypothesis-generating rather than as definitive evidence of superiority.

After external validation, the DVTS may facilitate risk characterization and research stratification; the present study does not support DVTS-guided modification of thromboprophylaxis, and no causal inference regarding prophylaxis use should be drawn. These risk categories may help define candidate groups for future prospective studies evaluating clinically relevant thromboembolic outcomes and selective imaging strategies; however, the present study was not designed to determine whether DVTS-guided management improves outcomes. This differentiated approach is consistent with recommendations from the American Society of Hematology and ESA/ESAIC guidelines emphasizing individualized risk assessment [22, 23]. 

### Simplified score and information loss

The conversion of continuous regression coefficients to integer points (0, 1, or 2) necessarily coarsens the prediction. The apparent AUC of the full logistic model was 0.83, compared with 0.81 for the simplified integer score — a reduction of 0.02 that represents the discrimination lost by simplification. To avoid conflating the two representations, the headline temporal-validation metrics reported throughout (AUC 0.82, calibration slope 0.94, calibration-in-the-large, Brier score, and decision curve analysis) were derived from the shrinkage-adjusted logistic model, whereas the integer DVTS is a bedside approximation with an apparent AUC of 0.81; the two are compared directly in Additional file 3. This trade-off between precision and bedside usability is common in clinical scoring systems. In this dataset, conversion of regression coefficients to integer points reduced AUC by 0.02, indicating a modest loss of discrimination in exchange for simpler bedside use. A detailed comparison of the full logistic model, shrinkage-adjusted model, and simplified integer score is provided in Additional file 3.

### Strengths and limitations

Strengths of this study include a relatively large consecutive cohort, temporal validation in an independent calendar-period sample, adherence to TRIPOD reporting principles, and standardized postoperative ultrasonographic surveillance. In the development cohort, 221 outcome events supported the final 12-predictor model.

Several limitations are central to interpreting these findings. First, this is a single-center, single-country study of standard-risk surgical femoral neck fracture patients (excluding those with recent VTE, active malignancy, or therapeutic anticoagulation); the score should not be applied to these excluded subgroups without further study, and while temporal validation supports transportability across time, generalizability to other institutions and populations requires external validation in multicenter settings. Second, ultrasonographers were not blinded to clinical information, which could introduce detection bias; specifically, clinicians aware of elevated D-dimer or prolonged immobilization may have performed more thorough examinations, potentially inflating DVT detection in high-risk patients and contributing to apparent model performance. Of the 269 DVT cases, 218 (81.0%) were detected on scheduled scans (24–48 h or day 7) and 51 (19.0%) on symptom-triggered imaging; model discrimination was similar when restricted to scheduled-scan DVT only (AUC 0.80, 95% CI: 0.76–0.84). Because outcome ascertainment combined scheduled scans and symptom-triggered imaging, calibration estimates may partly reflect differences in clinical detection intensity rather than only underlying thrombotic risk. Moreover, because the primary endpoint combined scheduled surveillance-detected and symptom-triggered DVT events, the model’s predictive value for proximal DVT or symptomatic VTE specifically remains to be established. Third, dichotomization of continuous predictors (age, D-dimer, albumin, CRP, platelets, surgery delay, operative time) improves bedside usability at the cost of information loss and may reduce transportability across settings and assays; for D-dimer in particular, the 500 ng/mL threshold is assay-dependent (FEU) and may require recalibration for other assay systems. Fourth, we did not evaluate genetic risk factors or novel biomarkers (tissue factor, P-selectin) that may further improve prediction [24]. Fifth, the 90-day follow-up period captures the majority of postoperative DVT events but may miss late cases. Sixth, early DVT diagnoses (Days 1–3, *n* = 90, 33.^5^% of events) may have included previously undetected preoperative thromboses because routine preoperative ultrasonography was not performed. In a sensitivity analysis excluding day-1 DVT cases (*n* = 12), the development AUC remained 0.81 (95% CI: 0.77–0.85), suggesting that pre-existing contamination did not materially alter overall discrimination, although residual misclassification cannot be excluded. Seventh, duration of thromboprophylaxis may have varied by clinician-perceived risk, as extended prophylaxis up to 35 days was at the discretion of the treating surgeon for patients with additional risk factors; this could have attenuated observed associations between predictors and DVT in higher-risk patients. Eighth, pulmonary embolism and major bleeding outcomes were not systematically captured in the source dataset and could not be reported reliably, and the predicted outcome (largely surveillance-detected DVT) is not concordant with current guidance, which does not endorse routine screening for subclinical DVT; the clinical actionability of the score is therefore limited and is discussed above. Ninth, the cost-effectiveness implications of DVTS-guided prophylaxis require formal health-economic evaluation with transparent reporting of perspectives, time horizons, utility values, and willingness-to-pay thresholds before definitive conclusions about economic impact can be drawn.

### Implications for clinical practice and research

In our inter-rater reliability study, 42 healthcare providers (exploratory reproducibility assessment) — including nurses who had received no formal training in DVT risk scoring — completed the DVTS calculation in under 3 minutes with substantial agreement (κ = 0.87; classified as ‘almost perfect’ agreement per Landis and Koch [25]). The absence of a significant difference between physician and nursing kappa values (*p* = 0.34) is important for implementation, as nursing-led DVT risk assessment is increasingly common in busy orthopedic wards where physician time is constrained. However, a prediction model should not be considered ready for routine implementation until it has been externally validated in at least one additional center.15 External validation studies in diverse settings, including different countries and healthcare systems, are the essential next step. Before routine use, the score should first be externally validated in at least one independent orthopedic cohort using comparable predictor definitions and surveillance methods.

The clinical role of the DVTS warrants careful framing. In this population, most patients already receive guideline-endorsed pharmacological thromboprophylaxis, and routine duplex screening for subclinical DVT is not currently recommended by major guidelines. Of the 269 events, 218 (81.0%) were detected during scheduled surveillance examinations and 51 (19.0%) on symptom-triggered imaging, and escalation of anticoagulation intensity in identified high-risk patients cannot be recommended on the basis of this study, particularly given the elevated bleeding risk in an elderly, frail population and because pulmonary embolism and major bleeding outcomes were not systematically captured in the source dataset and could not be reported reliably. Accordingly, the DVTS should not be interpreted as a tool to guide intensified anticoagulation in current practice. Its more realistic near-term value is to characterize the perioperative and laboratory risk profile of this population and, after external validation, to serve as an enrichment tool for future research — for example, to identify higher-risk subgroups for trials of targeted interventions (such as proximal DVT management strategies) or to enrich research cohorts evaluating selective imaging strategies and clinically relevant thromboembolic outcomes.

## Conclusion

We developed and temporally validated a 12-predictor shrinkage-adjusted logistic model and evaluated a derived simplified integer DVTS for DVT diagnosed after femoral neck fracture surgery. In this single-center study, the shrinkage-adjusted model showed good discrimination and acceptable calibration, while the integer DVTS stratified patients into low-, moderate-, and high-risk groups with observed DVT rates of 3.3%, 11.7%, and 29.2%, respectively. Because the outcome may have included previously undetected preoperative events and neither the model nor the simplified integer score has undergone external validation, both should be considered preliminary tools for DVT risk characterization rather than guides to intensified anticoagulation. External validation and prospective impact assessment are required before clinical application. 

## Supplementary Information

Below is the link to the electronic supplementary material.

Supplementary Material 1

Supplementary Material 2

Supplementary Material 3

## Data Availability

The datasets generated and analyzed during the current study are available from the corresponding author on reasonable request, subject to institutional data governance policies.

## Declarations

### Declarations of generative AI in scientific writing

During the preparation of this manuscript, the authors used a generative AI tool (Claude, Anthropic) only for language editing and improving readability. The authors reviewed, verified, and edited all AI-assisted output. No AI tool was used to generate or analyze data, create results, or draw scientific conclusions. The authors take full responsibility for the content of this manuscript.
