# Supplementary material for: Development and temporal validation of a clinical prediction score for deep vein thrombosis following femoral neck fracture surgery: a retrospective cohort study with decision curve analysis
Source: Thromb J. 2026 Jul 27;24:96. doi: 10.1186/s12959-026-00898-x (PMC13692907; doi:10.1186/s12959-026-00898-x)
Supplement: Supplementary file 1 [file 12959_2026_898_MOESM1_ESM.docx]

**Supplementary Table S2. Candidate Predictors Considered Before Model Fitting**

*All 25 candidate variables identified a priori from published literature and routine clinical records are listed below. Twelve predictors were selected before multivariable modeling for inclusion in the final pre-specified model. No data-driven stepwise selection was applied.*

| **Variable** | **Coding** | **Missing (%)** | **Clinical Rationale** | **In Final Model** | **Reason if Excluded** |
| --- | --- | --- | --- | --- | --- |
| Age >75 years | Binary | <0.1 | Established age-related VTE risk; consistent with ACCP/NICE guidelines | **Yes** | — |
| ASA score ≥3 | Binary | 0.2 | Guideline-level perioperative risk marker (ASH, ESAIC) | **Yes** | — |
| BMI >30 kg/m² | Binary | 0.8 | Obesity as independent VTE risk factor | **Yes** | — |
| Garden classification III/IV | Binary | 0.1 | Displaced fracture associated with greater tissue injury and immobility | **Yes** | — |
| D-dimer >500 ng/mL | Binary | 0.3 | Biomarker of coagulation activation; strong published association with DVT | **Yes** | — |
| Albumin <35 g/L | Binary | 0.4 | Marker of malnutrition/inflammation; predicts thrombosis in elderly surgical patients | **Yes** | — |
| CRP >5 mg/L | Binary | 0.5 | Acute-phase inflammatory marker | **Yes** | — |
| Platelet count >400×10⁹/L | Binary | 0.2 | Reactive thrombocytosis associated with thrombotic risk | **Yes** | — |
| Time to surgery >48 hours | Binary | 0.1 | Prolonged immobilization before surgery increases DVT risk | **Yes** | — |
| Operation duration >90 min | Binary | 0.3 | Prolonged surgery increases venous stasis and tissue factor exposure | **Yes** | — |
| Perioperative hypotension (MAP <65 mmHg >20 min) | Binary | 1.2 | Hemodynamic instability may impair venous return | **Yes** | — |
| Blood transfusion >2 units | Binary | 0.4 | Transfusion-related inflammation and hypercoagulability | **Yes** | — |
| Sex (female) | Binary | 0 | Potential VTE risk modifier | **No** | Not retained because incremental value beyond age and BMI-based stratification was considered limited for the intended bedside model |
| Hypertension | Binary | 0.1 | Cardiovascular comorbidity | **No** | Not a recognized independent DVT predictor in orthopedic trauma guidelines; excluded for clinical redundancy |
| Diabetes mellitus | Binary | 0.2 | Metabolic comorbidity | **No** | Excluded because thrombotic relevance was considered partly captured by BMI, frailty, and perioperative immobility-related variables |
| Cardiac disease | Binary | 0.3 | Cardiovascular comorbidity | **No** | Clinical overlap with ASA classification; excluded to avoid redundancy |
| Chronic kidney disease | Binary | 0.5 | Renal impairment may affect coagulation | **No** | Not routinely retained in fracture-specific bedside DVT models; excluded for parsimony and to preserve usability |
| Mean platelet volume >10.5 fL | Binary | 0.3 | Platelet activation marker | **No** | High collinearity with platelet count (VIF 3.2); retained platelet count >400 as more widely available and standardized marker |
| Fibrinogen (continuous) | Continuous | 1.1 | Coagulation factor | **No** | Collinear with D-dimer; D-dimer retained as a more established and widely used fibrin turnover marker |
| PT/aPTT/INR | Continuous | 0.6 | Coagulation profile | **No** | Reflects anticoagulant therapy status rather than intrinsic thrombotic risk; not clinically suitable as predictor in anticoagulated population |
| Hemoglobin (continuous) | Continuous | 0.2 | Anemia as frailty marker | **No** | Clinical information largely captured by the transfusion requirement variable; excluded to avoid redundancy |
| WBC count | Continuous | 0.2 | Inflammatory marker | **No** | Non-specific inflammatory marker with clinical overlap with CRP; CRP retained as more directly relevant |
| Intraoperative blood loss >500 mL | Binary | 1.8 | Surgical trauma severity | **No** | Highly collinear with transfusion requirement (r=0.74); transfusion retained as more reliably and consistently recorded |
| Type of anesthesia (regional vs general) | Binary | 0.1 | Anesthesia may affect venous hemodynamics | **No** | Potential reverse causation: anesthesia choice may be influenced by perceived DVT risk, making inclusion methodologically problematic |
| Surgical procedure type (fixation vs arthroplasty) | Binary | 0.1 | Procedure complexity | **No** | Confounded by fracture type and surgeon preference; not suitable as an independent predictor variable |

Variables above the line (n=12) were included in the final pre-specified model; variables below the line (n=13) were excluded before model fitting. VIF, variance inflation factor; MAP, mean arterial pressure; ACCP, American College of Chest Physicians; NICE, National Institute for Health and Care Excellence; ASH, American Society of Hematology; ESAIC, European Society of Anaesthesiology and Intensive Care.
