# Supplementary material for: Development and temporal validation of a clinical prediction score for deep vein thrombosis following femoral neck fracture surgery: a retrospective cohort study with decision curve analysis
Source: Thromb J. 2026 Jul 27;24:96. doi: 10.1186/s12959-026-00898-x (PMC13692907; doi:10.1186/s12959-026-00898-x)
Supplement: Supplementary file 2 [file 12959_2026_898_MOESM2_ESM.docx]

**Supplementary Table S1. TRIPOD Checklist for Prediction Model Development and Validation**

*TRIPOD = Transparent Reporting of a multivariable prediction model for Individual Prognosis Or Diagnosis. Items marked with page/section references indicate where each element is reported in the manuscript.*

| **Section/Topic** | **Item** | **Checklist Item** | **Reported on** |
| --- | --- | --- | --- |
| **Title** | 1 | Identify the study as developing and/or validating a multivariable prediction model, the target population, and the outcome to be predicted | Title, p.1 |
| **Abstract** | 2 | Provide a summary of objectives, study design, setting, participants, sample size, predictors, outcome, statistical analysis, results, and conclusions | Abstract, p.1–2 |
| **Background and objectives** | 3a | Explain the medical context and rationale for developing/validating the prediction model | Introduction, p.3–4 |
|  | 3b | Specify the objectives, including whether developing and/or validating | Introduction, p.4 |
| **Source of data** | 4a | Describe the study design or source of data | Methods 2.1, p.4 |
|  | 4b | Specify the key study dates, including start and end of accrual and follow-up | Methods 2.1–2.3, p.4–5 |
| **Participants** | 5a | Specify key elements of the study setting | Methods 2.1–2.2, p.4–5 |
|  | 5b | Describe eligibility criteria | Methods 2.2, p.5; Figure 1 |
|  | 5c | Give details of treatments received, if relevant | Methods 2.5, p.6 |
| **Outcome** | 6a | Clearly define the outcome | Methods 2.4, p.5–6 |
|  | 6b | Report actions to blind outcome assessment | Methods 2.4, p.6 (absence of blinding reported) |
| **Predictors** | 7a | Clearly define all predictors, including timing of measurement | Methods 2.6, p.6–7 |
|  | 7b | Report any actions to blind predictor assessment | N/A (retrospective) |
| **Sample size** | 8 | Explain how the study size was arrived at | Methods 2.8, p.7 |
| **Missing data** | 9 | Describe how missing data were handled | Methods 2.7, p.7 |
| **Statistical analysis** | 10a | Describe how predictors were handled in the analyses | Methods 2.9, p.7–8 |
|  | 10b | Specify type of model, all model-building procedures, and method for internal validation | Methods 2.9, p.7–8 |
|  | 10c | For validation, describe how predictions were calculated | Methods 2.9, p.8 |
|  | 10d | Specify all measures used to assess model performance | Methods 2.9, p.8 |
| **Risk groups** | 11 | Provide details on how risk groups were created | Results 3.4, p.10 |
|  | 12 | For validation, identify any differences from the development data in setting, eligibility criteria, outcome, and predictors | Methods 2.3, 2.5, p.5–6; identical eligibility criteria, ultrasound protocol, laboratory assays, and thromboprophylaxis protocol applied across both periods |
| **Participants** | 13a | Describe the flow of participants through the study | Results 3.1, p.9; Figure 1 |
|  | 13b | Describe the characteristics of the participants | Results 3.1, p.9; Table 1 |
| **Model development** | 14a | Specify the number of participants and outcome events | Results 3.1–3.2, p.9 |
|  | 14b | If done, report the unadjusted association between each candidate predictor and outcome | Results 3.3, p.9–10; Table 2 |
| **Model specification** | 15a | Present the full prediction model | Results 3.4, p.10; Tables 3–4; Figure 4 |
|  | 15b | Explain how to use the prediction model | Results 3.4, p.10; Figure 4 legend |
| **Model performance** | 16 | Report performance measures with CIs | Results 3.5–3.6, p.10–11; Table 6; Figures 5, 7 |
| **Updating** | 17 | If done, report the results from any model updating | Results 3.4, p.10 (shrinkage adjustment as model recalibration) |
| **Discussion** | 18 | Discuss any limitations of the study | Discussion, Limitations, p.13 |
|  | 19a | Discuss the results in context of existing models | Discussion, p.12–13 |
|  | 19b | Give an overall interpretation of results considering objectives, limitations, and similar studies | Discussion, p.12–14; Conclusion, p.14 |
| **Supplementary** | 20 | Provide information about the availability of supplementary resources | Declarations, p.14 |
|  | 21 | Discuss the potential clinical use of the model and implications for future research | Discussion, p.12–14; Conclusion, p.14 |
| **Funding** | 22 | Give the source of funding and the role of funders | Declarations, p.14 |

*Adapted from Collins GS, Reitsma JB, Altman DG, Moons KG. Transparent Reporting of a multivariable prediction model for Individual Prognosis Or Diagnosis (TRIPOD): the TRIPOD statement. Ann Intern Med. 2015;162(1):55-63.*
