# Supplementary material for: Development and temporal validation of a clinical prediction score for deep vein thrombosis following femoral neck fracture surgery: a retrospective cohort study with decision curve analysis
Source: Thromb J. 2026 Jul 27;24:96. doi: 10.1186/s12959-026-00898-x (PMC13692907; doi:10.1186/s12959-026-00898-x)
Supplement: Supplementary file 3 [file 12959_2026_898_MOESM3_ESM.docx]

**Supplementary Table S3. Performance Comparison: Full Logistic Model vs. Shrinkage-Adjusted Model vs. Simplified Integer Score**

| **Metric** | **Full Logistic Model (apparent)** | **Shrinkage-Adjusted Model** | **Simplified Integer Score** |
| --- | --- | --- | --- |
| **Development Cohort (n=1,829)** |  |  |  |
| AUC (95% CI) | 0.83 (0.79–0.87) | 0.83 (0.79–0.87) | 0.81 (0.77–0.85) |
| Calibration slope | 1.00 | 1.00 (by design) | Not separately estimated |
| CITL | 0.00 | 0.00 (re-estimated) | Not separately estimated |
| Brier score | 0.084 | 0.087 | Not separately estimated |
|  |  |  |  |
| **Temporal Validation (n=412)** |  |  |  |
| AUC (95% CI) | 0.82 (0.77–0.87) | 0.82 (0.77–0.87) | 0.81 (0.76–0.86) |
| Calibration slope (95% CI) | 0.90 (0.85–0.95) | 0.94 (0.89–0.99) | Not separately estimated |
| Brier score | 0.085 | 0.084 | Not separately estimated |

Full logistic model: all 12 predictors with original (unshrunk) regression coefficients. Shrinkage-adjusted model: coefficients multiplied by uniform shrinkage factor (0.92) with intercept re-estimated. Simplified integer score: binary point allocation (0, 1, or 2 points per predictor) derived from shrinkage-adjusted coefficients. The AUC reduction from the full model to the integer score (0.83 → 0.81 in development and 0.82 → 0.81 in temporal validation) quantifies the modest discrimination cost of score simplification.

*Because a uniform shrinkage factor multiplies all coefficients by the same constant, it preserves the rank order of the linear predictor; the full and shrinkage-adjusted logistic models therefore have identical AUCs, differing only in calibration. Bootstrap optimism-corrected AUCs were 0.80 (shrinkage-adjusted logistic model) and 0.79 (simplified integer DVTS). Calibration, CITL, and Brier score were not separately estimated for the non-recalibrated integer score. CITL, calibration-in-the-large.*
